# Heart-brain interactions: clinical evidence and mechanisms based on critical care medicine

**DOI:** 10.3389/fcvm.2024.1483482

**Published:** 2024-11-29

**Authors:** Chuyao Qi, Wenting Wang, Yanfei Liu, Tianfeng Hua, Min Yang, Yue Liu

**Affiliations:** ^1^ Laboratory of Cardiopulmonary Resuscitation and Critical Care, The Second Affiliated Hospital of Anhui Medical University, Hefei, Anhui, China; ^2^ National Clinical Research Center for TCM Cardiology, Xiyuan Hospital of China Academy of Chinese Medical Sciences, Beijing, China

**Keywords:** heart-brain interaction, critical care medicine, intensive care unit, clinical evidence, mechanism, progress

## Abstract

In this review paper, we search the recent literature regarding the application of the heart-brain interaction theories in the field of intensive care unit. Simultaneously, we methodically summarize the clinical evidence supporting its application in intensive care unit treatment, based on clinical randomized trials and clinical case studies. We delve into how it's applied in treating severely ill patients and in researching animal models for cardio-cerebral comorbidities, aiming to supply benchmarks for subsequent clinical trials and studies on mechanisms.

## 1 Introduction

According to the World Health Organization, cardio-cerebrovascular diseases, such as stroke and myocardial infarction, have remained the primary reason for mortality and disability worldwide for the last 15 years (1). Growing studies in the field of cardio-cerebrovascular diseases have revealed a significant connection between cardiovascular diseases and neurological conditions, leading to a new discipline known as “Neurocardiology”. A research indicates that a 50%–100% surge in both stroke survivors and patients over the past 25 years, with a notable rise in the disease burden of patients (2, 3), potentially linked to cardiac issues caused by stroke. Stroke ranks as the second most prevalent cause of mortality globally (4). A range of new-onset cardiovascular complications, such as arrhythmias and takotsubo cardiomyopathy, may occur subsequent to a stroke (5). The cardiac death rate is elevated (19.4% of all deaths) during the initial 4 weeks after stroke (6). Concurrently, the management of neurological disorders like acute ischemic stroke (AIS) faces significant hurdles due to cardiac complications (7, 8). Meanwhile, heart failure (HF) represents a significant health challenge globally and ranks as a primary reason for unfavorable outcomes and death rates (9).

Historically, academic studies have concentrated on the distinct therapeutic approaches of cardiology and neurology for cardio-cerebrovascular diseases management. Consequently, a deficit exists in the systematic and uniform interdisciplinary foundational and clinical studies across various disciplines concerning cardio-cerebrovascular disorders. Furthermore, the engagement of intensive care physicians is essential for patients in critical conditions (10). This review searches both fundamental and clinical researches concerning the use of heart-brain interaction theories in critical care, aiming to encapsulate the latest clinical research findings on its application in preventing and treating severe cardio-cerebrovascular diseases, along with its effect mechanisms, to guide future pertinent studies.

## 2 The theories of heart-brain interaction

**Figure 1:**
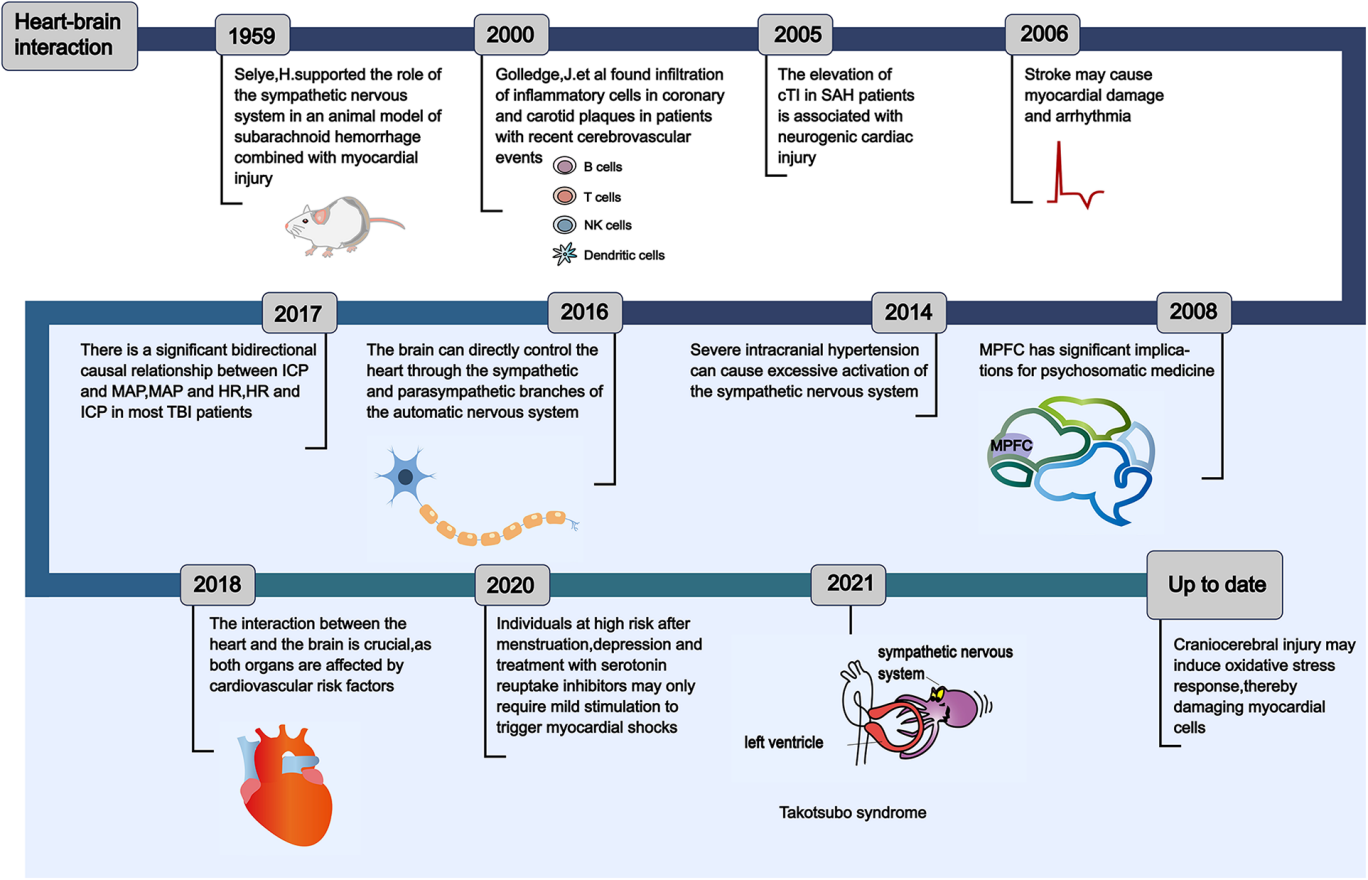
The historical evolution of the concept of heart-brain interaction. This reveals the evolution process of the theories of heart-brain interaction, which has been understood and discovered by scholars from different perspectives from ancient times to the present. Understanding the evolution of the theories of heart-brain interaction can provide important insights for the prevention of critical cardio-cerebrovascular diseases in intensive care unit. cTI, cardiac tropnin I; SAH, subarachnoid hemorrhage; MPFC, medial prefrontal cortex; ICP, intracranial pressure; MAP, mean arterial pressure; HR, heart rate; TBI, traumatic brain injury.

Numerous theories regarding heart-brain interaction have emerged over the past hundred years, influenced by clinical symptoms and the progression of pathological alterations (Figure 1). This section will present the heart-brain interaction concepts from two perspectives: fundamental scientific research experiments and clinical case studies. In 1959, Selye (11) developed an animal model of subarachnoid hemorrhage (SAH) and used drugs like reserpine, which block catecholamine release, to avert myocardial damage. This research indicated that cardiac necrosis following SAH is attributable to catecholamine toxicity. Furthermore, it demonstrated that catecholamines released directly into the heart via the nervous system exhibit significantly greater toxicity compared to those that arrive at the heart through the bloodstream. Subsequently, Masuda et al. (12) observed that in the acute phase of SAH (180 min after SAH), heightened sympathetic nervous system (SNS) activity has been associated with myocardial injury and contributes to the development of cardiac dysfunction. Thackeray et al. (13) employed serial noninvasive whole-body positron emission tomography to concurrently interrogate the heart and the brain. The positron emission tomography imaging agents utilized in this study were specifically designed to target the 18-kDa mitochondrial translocator protein, which is known to be upregulated in activated microglia and in systemic monocytes. Inflammatory responses are observed in both the brain and the heart one week following an acute myocardial infarction (AMI).

**Figure 2:**
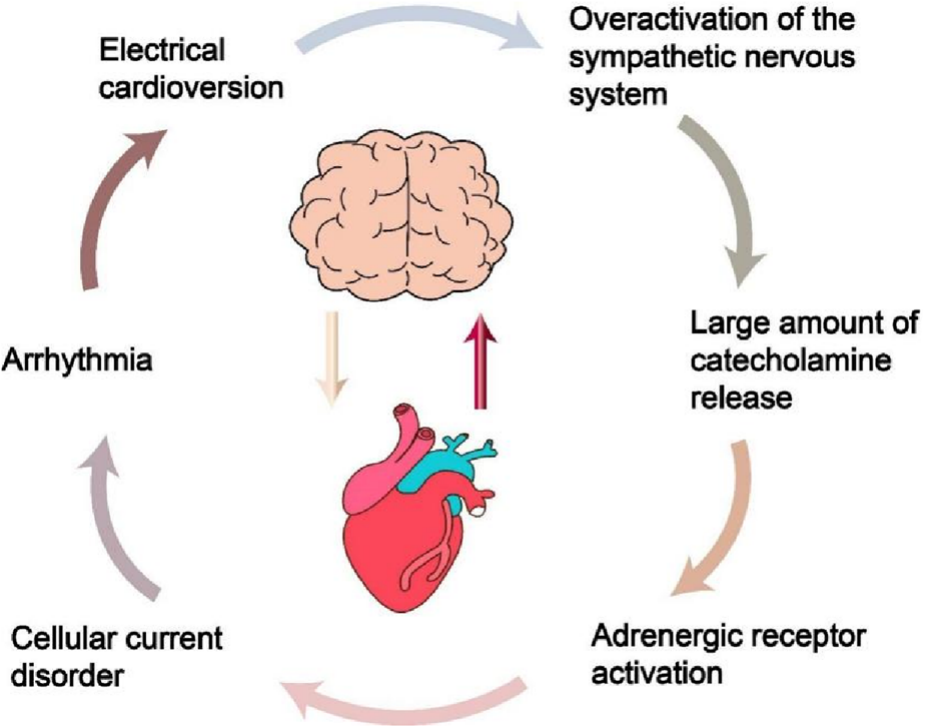
The process of intracranial hypertension leading to heart damage. Understanding this process can provide a basis for preventing cardiovascular complications in patients with traumatic brain injury in intensive care unit.

A research in 2014 revealed that intense intracranial hypertension, such as SAH, excessively stimulates the SNS (Figure 2), leading to the development of serious cardiac arrhythmia in patients (14). Following this, Silvani et al. (15) found that the brain innervates the heart via the sympathetic and parasympathetic pathways of the autonomic nervous system (ANS), and neurological conditions may disrupt the central autonomic regulation of the cardiovascular system, resulting in grave outcomes like myocardial injury, arrhythmia, and potentially abrupt mortality. During the year 2018, Doehner and colleagues (16) clearly suggested that the heart and the brain interact in two directions. For instance, individuals diagnosed with severe HF exhibit a diminished left ventricular ejection fraction (LVEF), which correlates with an elevated annual stroke risk estimated at 4%. In contrast, patients experiencing mild to moderate HF have an approximate annual stroke risk of 1.5%. A sympathetic-vagal imbalance due to stroke may lead to a decrease in myocardial contractility and trigger the lysis of myocardial cells. The aforementioned studies indicate that the central nervous system (CNS)' s control over heart function is attainable via the outflow of cardiac motor sympathetic and parasympathetic signals to both the cardiac conduction system and the myocardium. Conversely, impairments in heart performance may impact the health of the brain, including neuroinflammation and the deterioration of cognitive functions. Neuroinflammation is characterized as the immune response elicited by the CNS in reaction to localized injury or systemic infection. Microglia is the main agonist of neuroinflammation and it is present in significant quantities within the brain (17). In addition, following heart transplantation or cardiac resynchronization therapy (CRT), there is an observed increase in cerebral blood flow and an enhancement in cognitive function among patients with severe HF. This observation suggests that the cognitive impairments observed in individuals with severe HF may be linked to the underlying mechanisms associated with impaired cardiac systolic function, which leads to a reduction in cardiac output. Such a decrease in output may result in diminished cerebral blood flow and subsequent cerebral ischemia due to insufficient cerebral perfusion (18–20).

To sum up, numerous academics have explored the heart-brain interaction through diverse viewpoints, formulated several innovative theories, and unearthed a plethora of fresh research findings, laying a robust groundwork for averting secondary brain injuries in patients with severe cardiovascular conditions (such as severe HF or cardiac dysfunction requiring mechanical cardiac support leading to cognitive impairment) or secondary cardiac issues in those with critical neurological conditions (such as severe stroke causing myocardial injury, arrhythmia, and potentially abrupt mortality) within intensive care unit (ICU). In the subsequent chapters, we will respectively elaborate on the application of the heart-brain interaction concepts within the contexts of clinical practice and fundamental scientific research experiments.

## 3 Heart-brain interactions: clinical evidence

### 3.1 Heart to brain: heart failure on cognitive function

Cardio-cerebrovascular diseases have consistently been a primary reason for hospital admissions, illness and death globally (1). The cardiovascular system plays a crucial role in circulating blood throughout the body, brain included. HF can result in neurological disorders like stroke, with Table 1 encapsulating the risk factors linked to stoke, HF included. Furthermore, it's proposed that diminished cardiac output could primarily lead to prolonged cerebral hypoperfusion and cognitive deterioration in patients suffering from HF (26, 27).

**Table 1:** Heart related risk factors for stroke.

Factors	Characteristic	Ref.
HF (heart failure)	There is an interaction between HF and stroke, and stroke increases the incidence rate and mortality of HF patients	(21)
Atrial fibrosis	Atrial fibrosis is the main cardiac risk factor for stroke	(22)
Hypertension	Hypertension is a common risk factor for recurrent stroke	(23)
Rheumatic heart disease	Rheumatic heart disease is one of the main causes of stroke in middle-and low-income countries	(24)
Valvular heart disease	American residents with valvular heart disease have a higher risk of experiencing stroke	(25)

Given that a malfunctioning heart can impair the standard structure and functionality of the brain and prompt treatment of the heart performance in severe HF patients can avert neurological conditions like stroke (28). The Montreal Cognitive Assessment serves as a diagnostic instrument for identifying cognitive impairment. Bhat et al. (29) conducted a follow-up study on patients with end-stage HF who had undergone implantation of left ventricular assist devices for a duration of eight months. The study revealed notable enhancements in the patients' The Montreal Cognitive Assessment total score, visuospatial, executive, and delayed recall cognitive domains. These findings indicated that improvements in cardiac function, characterized by reductions in mitral and tricuspid regurgitation and decreased serum levels of B-type natriuretic peptide, are associated with positive effects on cognitive function in the brain. Research conducted by Lee and colleagues also showed that individuals with advanced HF exhibit atypical brain metabolism, characterized by significantly reduced creatine levels in the parietal white matter compared to control subjects. Notably, these cerebral metabolic abnormalities can be ameliorated following an enhancement in LVEF subsequent to heart transplantation (30). Bommel et al. (31) conducted a thorough investigation into how cardiovascular system operations impact the nervous system. Patients with HF were randomly divided into two groups. Patients in the control group received medication and lifestyle enhancements due to their unsuitability for CRT, in contrast to the treatment group who underwent a 6-month CRT. The results showed that enhanced systolic function in the left ventricle of the treated group leads to a rise in cerebral blood circulation and better cognitive abilities in the patients. A clinical trial was conducted involving patients with life-threatening, terminal HF. The researchers objectively assessed neurocognitive function by utilizing cognitive P300 auditory evoked potentials. The results demonstrated that after the successful implantation of a ventricular assist device, there was a notable increase in the P300 evoked potential in patients suffering from severe HF, alongside an enhancement in neurocognitive function (32). Additionally, a separate investigation into patient suffering from severe HF revealed that enhancements in LVEF achieved via CRT are associated with improvements in cognitive impairment among patients, specifically in the executive functioning, global cognition, and visuospatial functioning (33).

To sum up, for severe HF patients, ameliorating heart performance proactively averts neurological disorders. Facing with complications like stroke or cognitive impairment, concurrent treatments of the heart and the brain are crucial for ameliorating the function of patients' heart and brain.

### 3.2 Brain to heart

#### 3.2.1 ANS dysfunction in takotsubo cardiomyopathy

Disorders related to neurology, such as AIS, SAH represent a primary contributing factor to the development of takotsubo cardiomyopathy (34). A substantial body of evidence indicates that takotsubo cardiomyopathy is associated with the heart-brain axis (35–37). Researchers administered β- blockers to patients with aneurysmal subarachnoid hemorrhage (aSAH) prior to their admission and utilized echocardiography to assess any impairment in ventricular systolic function. The findings indicated that the intervention group exhibited a significantly reduced incidence of takotsubo cardiomyopathy following aSAH in comparison to the control group. This study primarily examined the application of β- blockers in patients with aSAH prior to hospital admission, focusing on preventive measures. However, there exists a paucity of clinical research data on this topic. The relationship between the risk of hypotension associated with β- blocker use and the objectives of SAH management post-aneurysm repair is inconsistent. Specifically, the management protocols may permit or, in certain instances, necessitate hypertension to mitigate the risk of cerebral vasospasm, which can lead to infarction. Consequently, it remains unclear whether the administration of β- blockers within 3 days after admission to reduce cardiac output in aSAH patients is harmful to the patients or beneficial in the pathogenesis of takotsubo cardiomyopathy caused by aSAH. Furthermore, the use of β- blockers is contraindicated in patients with acute and severe HF who exhibit low LVEF, hypotension, and in those with bradycardia (38).

Table 2 encapsulates the progress made in studying the link between takotsubo cardiomyopathy and CNS disorders. Scholars have discovered that enhancements in cardiac function, including improvements in cardiac contraction, can lead to amelioration of cognitive impairments. Conversely, the restoration of CNS function, exemplified by the inhibition of excessive SNS activation, has been shown to positively influence cardiac function. This includes benefits such as improved myocardial remodeling, a reduction in arrhythmias and the prevention of sudden cardiac death. Research indicates that alterations in ECG among patients with takotsubo cardiomyopathy frequently manifest within 10 h following AIS events. Notably, the majority of these ECG changes (including ST-segment elevation and negative giant T waves) are not associated with cardiac symptoms, which are typically absent upon admission. Additionally, echocardiographic assessments reveal localized dysfunction in LV motility, particularly in the apical region (43). The ANS, which includes nucleus ambiguus, the nucleus tractus solitarius, the dorsal motor nucleus of the vagus, and the rostral ventrolateral medulla, receives regulatory information from the CNS and exerts control over the heart, blood vessels, and adrenal glands via pressure receptors. Consequently, this study posits that significant ischemia in the brainstem may result in disorders of the ANS, potentially contributing to the development of takotsubo cardiomyopathy. Patients with takotsubo cardiomyopathy who present with elevated intraventricular pressure gradients (PG) attributed to heightened SNS activation in a clinical trial were divided into two distinct groups. Participants in the intervention group received propranolol, a β-blocker more than 24 h following the onset of the condition. Meanwhile, those in the control group complied with clinical treatment guidelines, such as lifestyle enhancements. Results suggested that the intervention group experienced a reduction in the intraventricular PG alongside an enhancement in LVEF. This suggests that β-blockers may confer cardioprotective effects by counteracting the overactivation of the SNS, diminishing the release of catecholamines and mitigating their deleterious effects on cardiac function (48). Nevertheless, several studies indicate that the intravenous administration of propranolol requires meticulous monitoring due to numerous contraindications associated with the use of β-blockers. These contraindications include conditions such as asthma, respiratory failure, diabetic coma, decompensated HF, vasospasm, and bradyarrhythmia (49).

**Table 2:** Central nervous system disorders and takotsubo cardiomyopathy: a historical perspective.

Year	Author	Description	Ref.
1991	Dote, K. et al.	First report takotsubo cardiomyopathy	(39)
1998	Brandspiegel, H.	Takotsubo cardiomyopathy is mainly caused by psychological factors	(40)
2005	Wittstein, I. S. et al.	The main reason may be overstimulation of the sympathetic nervous system	(41)
2007	Dorfman, T. et al.	Takotsubo cardiomyopathy maybe is induced by multiple sources of physical stress	(42)
2008	Yoshimura, S. et al.	Takotsubo cardiomyopathy can be caused by acute ischemic stroke	(43)
2014	Suzuki, H. et al.	The role of brain cardiac axis in the pathogenesis of takotsubo cardiomyopathy is crucial	(44)
2015	Templin, C. et al.	Takotsubo cardiomyopathy is induced by acute, past, or chronic neurological or psychiatric disorders	(36)
2016	Ghadri, J. R. et al.	Neurological disorders may lead to morphological changes in the heart	(45)
2020	Amin, H. Z. et al.	High levels of catecholamines play a crucial role in the pathogenesis and pathophysiology of takotsubo cardiomyopathy	(46)
2022	Matta, A. et al.	Sympathetic nerve stimulation and catecholamine storm play a central role in the development of takotsubo cardiomyopathy	(47)

Currently, there is a lack of targeted intervention strategies for the management of takotsubo cardiomyopathy in patients suffering from severe cerebrovascular conditions, including aSAH, and AIS. Consequently, it is prudent to conduct further investigations into the incidence of takotsubo cardiomyopathy both prior to and following hospital admission, as well as to determine the optimal timing for interventions, appropriate dosages, and the impact of such treatments on cardiac and neurological function in this patient population, particularly with respect to the use of β-blockers. Future clinical randomized controlled trials may provide valuable insights that could enhance cardiac function—addressing issues such as left ventricular outflow tract obstruction, cardiogenic shock, and arrhythmias—by facilitating the recovery of compromised CNS functions, which may include addressing cerebral tissue ischemic infarction, secondary cerebral vasospasm resulting from elevated intracranial pressure, acute cerebral edema, and extensive neuronal death.

#### 3.2.2 Systematic immunity

**Figure 3:**
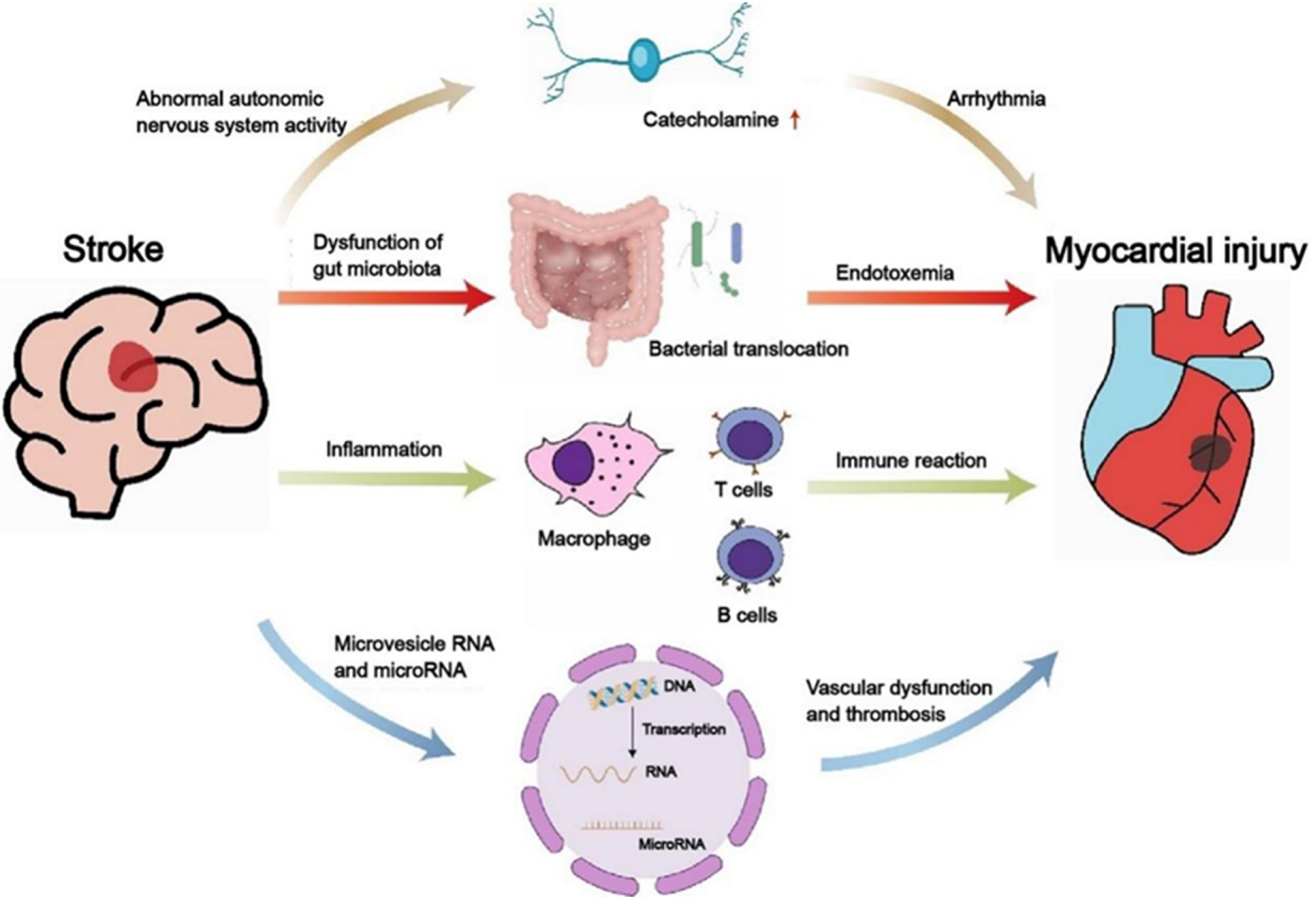
The relationship between stroke and cardiac dysfunction. Displayed the mechanisms of the activity of the autonomic sympathetic nervous system, immune system, gut microbiome, and microRNA and microvesicle RNA. Clarifying the interaction between heart and brain will help develop more appropriate and effective treatment strategies for severely ill patients with cardio- cerebrovascular diseases.

The pathological mechanisms of heart dysfunction caused by acute cerebrovascular disorders remain unelucidated, potentially linked to malfunctions in the ANS, intestinal microbiota, inflammation, microvesicles and microRNAs (Figure 3). Intervening in the aforementioned mechanisms can avert cardiovascular complications in patients suffering from severe cerebrovascular accidents. The benefits and drawbacks of intervening in ANS dysfunction resulting from acute cerebrovascular accidents (including aSAH and AIS) to prevent or address cardiac dysfunction (such as reduced ventricular systolic function and LVEF) in patients have been thoroughly examined in previous literature. Consequently, this discussion will primarily concentrate on the local and systemic inflammatory responses observed in both the brain and the heart. Bilt and colleagues (50) discovered that patients with SAH exhibit myocardial damage and neutrophils are present in this region. Levels of Tumor Necrosis Factor-alpha (TNF-α) escalate in correlation with the severity of cardiovascular diseases. Research has demonstrated that anti-TNF-α therapy can enhance cardiac function in patients with HF. This improvement is attributed to the phenomenon whereby stress-activated cytokines in cardiac tissue surpass the capacity of local cell receptors involved in autocrine and paracrine signaling, subsequently entering the systemic circulation as hematogenous cytokines. These cytokines, being large molecules, typically face challenges in crossing the blood-brain barrier. However, in instances where the blood-brain barrier surrounding the periventricular organs is compromised or absent, these cytokines can infiltrate the brain via saturable or passive transport mechanisms. Once in the brain, they may act as mediators of inflammation and stimulate the SNS. Nevertheless, existing research has yet to elucidate the precise mechanisms underlying the interplay between cardiac injury and neuroinflammation. Consequently, additional experimental studies and clinical trials are warranted to further investigate and validate these interactions in the future.

## 4 The animal models for cardio-cerebral co-morbidities

The preceding section has provided an overview of the utilization of the heart-brain interaction concepts in the prevention and management of critically ill patients suffering from cardio-cerebrovascular diseases within clinical settings. This section will concentrate on presenting certain heart-brain interaction concepts that have been identified through animal studies but have not been implemented in actual clinical practice. Chen and colleagues (51) developed a test model for AIS using adult mice free from primary heart conditions or pre-existing vascular lesions. The results indicated that mice exhibit heart irregularities, including a decrease in cardiac ejection fraction, enlargement of cardiomyocytes and myocardial fibrosis. Subsequently, Bieber and colleagues (52) observed a reduction in heart rate, an enhancement in ejection fraction, and a notable decline in plasma brain natriuretic peptide levels in AIS mice following the administration of β- blockers. These findings indicate that β-blockade may mitigate the progression of chronic cardiac dysfunction by decelerating cardiac remodeling and suppressing sympathetic nerve activity linked to chronic autonomic dysfunction. Another study revealed that in a traumatic brain injury model, mice brain tissues produce microvesicles, potentially linked to increased blood coagulation (53). Research indicates that following injury to the left insular cortex in murine models, there is a notable decrease in both left ventricular systolic pressure and left ventricular end diastolic pressure, accompanied by elevated levels of norepinephrine in both plasma and myocardial tissues. These findings imply that focal ischemia in the left hemisphere of the brain may contribute to cardiac dysfunction, with the extent of this dysfunction correlating with the severity of damage to the insular cortex. Furthermore, the excessive release of catecholamines may play a mediating role in the observed cardiac dysfunction (54). Following a successful induction of selective insular cortex AIS in rats over a period of 28 days, Balint et al. (55) conducted a histological analysis that revealed a pronounced severity of endothelial dysfunction, inflammation, and fibrosis in the left atrial tissue adjacent to the pulmonary vein. This region is characterized by the highest concentration of sympathetic nerve processes within the atrial myocardium. This study posited that excessive activation of endothelial cells within the brain may facilitate the swift infiltration of neutrophils into myocardial tissue via the vascular endothelium, highlighting the possible involvement of autonomic dysfunction in the onset of cardiac injury. Nevertheless, additional research is warranted to uncover novel targets aimed at preventing structural alterations in the left atrium resulting from stroke, as well as addressing potential post-stroke cardiovascular complications, including arrhythmias and sudden cardiac death. Future investigations should also concentrate on the temporal evolution of left atrial heart disease triggered by ischemic strokes, alterations in the ventricles and right atrium, and myocardial changes that occur following infarcts of comparable size in other cerebral regions.

Francis et al. (56) observed an elevation in the synthesis of TNF-α within the brain, heart, and plasma of rats following AMI. This finding implies that AMI may trigger the production of pro-inflammatory cytokines in the hypothalamus. Concurrently, Meissner et al. (57) proposed that the administration of pharmacological agents that inhibit pro-inflammatory TNF-α in HF murine models markedly mitigated the reduction in dendritic spine density and the associated memory deficits induced by HF. Research has demonstrated that anti-TNF-α therapy can enhance cardiac function in rats with HF. This improvement is attributed to the phenomenon whereby stress-activated cytokines in cardiac tissue surpass the capacity of local cell receptors involved in autocrine and paracrine signaling, subsequently entering the systemic circulation as hematogenous cytokines. These cytokines, being large molecules, typically face challenges in crossing the blood-brain barrier. However, in instances where the blood-brain barrier surrounding the periventricular organs is compromised or absent, these cytokines can infiltrate the brain via saturable or passive transport mechanisms. Once in the brain, they may act as mediators of inflammation and stimulate the SNS. Consequently, anti-TNF-α therapy may enhance LVEF by mitigating the levels of cytokines and oxidative stress within the brain, ultimately leading to improved cardiac function in rats (58). Currently, numerous innovative strategies have emerged for the prevention and treatment of myocardial inflammation, particularly concerning injury and repair mechanisms. Among these is the identification of a novel specific myocardial cell within the damaged heart, as well as the Ym-1hi Neu subpopulation of cardiac neutrophils, which exhibit functional heterogeneity (59). Table 3 provides a summary of various animal models along with their respective features.

**Table 3:** Preparation of animal models for cardio-cerebrovascular diseases.

Animal model	Examples	Characteristic
Traumatic brain injury	Mouse model of fluid percussion injury (60)	With the heads of mice unconstrained, rapidly injecting saline into the closed skull cavity of the mice's heads under controlled pressure of 1.9 ± 0.1 atmospheres Effective, reliable, easy to operate, and clinically relevant
	Extraluminal Permanent distal middle cerebral artery occlusion Model (51)	A midline incision is opened between the orbit and the ear Clear penumbra can be seen around the infarcted core Animals have a longer survival time and small infarct area (61–63)
Acute myocardial infarction	The model of acute myocardial infarction in rats by ligation of coronary artery (64)	Ligation of the left anterior descending branch of the left coronary artery in rats Similar to the actual pathogenesis of myocardial infarction
Takotsubo cardiomyopathy	Rat model of takotsubo cardiomyopathy *in vivo* (65)	Injecting adrenaline into the external jugular vein Easy to operate

## 5 Conclusion and perspective

Managing cardio-cerebrovascular diseases clinically stands as a significant worldwide issue in public health (66). Approximately 1.6% of patients suffering from AIS experience cardiac complications like AMI, leading to a significant rise in both the hospital mortality rate and healthcare expenses for these patients (67). Meanwhile, HF poses a major risk for the emergence of AIS. Strategies like intensive heart rate management and antithrombotic interventions markedly lower stroke occurrences in HF patients, leading to a substantial decrease in hospital admissions and death rates (68). The central autonomic network can link with the intracardiac nervous system through the exogenous CNS, issuing instructions to the cardiac conduction system that are subsequently relayed to the myocardium (69). Yet, in states of sleep or emotional stimulation, the central autonomic network stimulates the heart through sympathetic and vagal nerves and is modulated by responses from myocardial and cardiovascular stress receptors (70).

The effect mechanisms of averting cardio-cerebrovascular diseases in severely ill patients, steered by the theories of heart-brain interaction, is linked to enhancing heart performance, improving ANS dysfunction and immune system regulation. Consequently, it acts as a directive for the prevention and management of severely ill patients suffering from takotsubo cardiomyopathy due to cerebrovascular diseases, severe HF coupled with neurological disorders. Concurrently, there are certain constraints associated with the outcomes of this review: regarding clinical evidence, the majority of literature fail to explicitly mention the individualized care of acute cardio-cerebrovascular patients within ICU. This includes considerations such as the adverse cardiac events associated with aSAH and the potential application of β- blockers during the acute phase of takotsubo cardiomyopathy resulting from acute cerebrovascular incidents. Further investigation is required into how ICU, the department of cardiovascular medicine and neurology department collaborate on cardio-cerebrovascular diseases. Moreover, there's a scarcity of clinical randomized controlled trials in ICU focusing on heart-brain interaction theories to direct the prevention and treatment of diverse cardio-cerebrovascular diseases, and the overall quality is subpar, missing in comprehensive, multi-center, large-sample randomized controlled trials. Regarding the study of effect mechanisms, the research on how the theories of heart-brain interaction guide the treatment of severely ill patients with cardio-cerebrovascular diseases is limited and there is a single research method.

In summary, prior research in ICU has laid a theoretical groundwork for preventing and treating acute cardio-cerebrovascular diseases, a frequent clinical crisis. A thorough examination of the theoretical underpinnings of these diseases can lead to innovative approaches in managing and preventing acute cardio-cerebrovascular diseases in severely ill patients. In my opinion, future research should prioritize the execution of fundamental experiments and the accumulation of clinical evidence to elucidate the interactions between the autonomic nervous system and cardiac function, as well as the relationship between cardiac injury and neuroinflammation. Furthermore, it is essential to enhance collaboration among the ICU, the Department of Cardiovascular Medicine and Neurology Department in the management and prevention of acute cardio-cerebrovascular diseases. The adoption of more advanced and precise technological methodologies is also recommended to thoroughly investigate potential novel mechanisms underlying the interaction between the heart and the brain. This will establish a link between fundamental principles and clinical practice, and lay the groundwork for the uniform application of heart-brain interaction theories in both preventing and treating severely ill patients.
